# Prediction of antibiotic resistance by gene expression profiles

**DOI:** 10.1038/ncomms6792

**Published:** 2014-12-17

**Authors:** Shingo Suzuki, Takaaki Horinouchi, Chikara Furusawa

**Affiliations:** 1 Quantitative Biology Center (QBiC), RIKEN, 6-2-3 Furuedai, Suita, Osaka 565-0874, Japan

## Abstract

Although many mutations contributing to antibiotic resistance have been identified, the relationship between the mutations and the related phenotypic changes responsible for the resistance has yet to be fully elucidated. To better characterize phenotype–genotype mapping for drug resistance, here we analyse phenotypic and genotypic changes of antibiotic-resistant *Escherichia coli* strains obtained by laboratory evolution. We demonstrate that the resistances can be quantitatively predicted by the expression changes of a small number of genes. Several candidate mutations contributing to the resistances are identified, while phenotype–genotype mapping is suggested to be complex and includes various mutations that cause similar phenotypic changes. The integration of transcriptome and genome data enables us to extract essential phenotypic changes for drug resistances.

## Abstract (web summary)

The relationship between mutations and phenotypic changes associated with drug resistance in bacteria remains unclear. Here, the authors use antibiotic-resistant *E. coli* strains, obtained by laboratory evolution, to show that resistance profiles can be predicted by changes in expression of a few genes.

## Graphical abstract

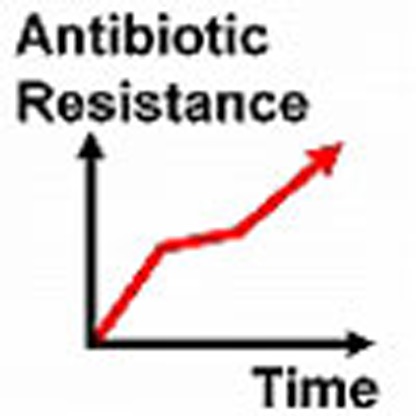

The emergence of multi-drug-resistant bacteria is a growing concern for global public health^1^,^2^,^3^, as doses of antibiotics have conferred a selective advantage for naturally emerged-resistant bacteria to cause drug ineffectiveness^4^,^5^. A number of mutations have been identified and shed light on how bacterial cells acquire antibiotic resistance^6^,^7^. For some of these mutations, scientists can easily extract the causal relationship to drug resistance, such as a resulting modification in a specific drug target^8^. However, the relationship between a mutation and drug resistance is not always a simple one-to-one correspondence. Multiple mutations are often required to acquire high levels of resistance to a specific drug^7^,^9^,^10^, whereas a single mutation can cause various phenotypic changes that change the resistance and susceptibility to various drugs simultaneously^11^. Studies using mutant libraries have revealed that a large number of genes influence drug resistance and susceptibility, including many genes not directly involved in known drug-resistant machineries^12^,^13^,^14^. Furthermore, non-additive (for example, synergistic and antagonistic) responses to combinatorial drug treatments suggest interplay among the mechanisms of drug resistances^15^,^16^. Overall, the complex relationship between drug resistance acquisition, genetic alternations and global phenotypic changes remains unclear.

Laboratory evolution of bacteria^17^ is a powerful tool for investigating the acquisition dynamics of drug resistance^7^,^18^. In such experiments, bacterial cells are exposed to fixed drug concentrations around which the cell growth is partially or completely inhibited such that a selective advantage for resistant strains is maintained. Although some essential factors in drug resistance evolution, including horizontal gene transfer (HGT)^19^ and interspecies communication^20^, are difficult to analyse using laboratory evolution, this experimental system has several advantages in comparison with *in vivo* experiments when studying *de novo* acquisition of drug resistance, including a well-characterized ancestor strain, a defined environment and parallel evolution experiments that discriminate necessary and unnecessary phenotypic/genetic changes. In general, the genome-wide phenotypic and genotypic analysis of emerging resistant strains in laboratory evolution offers to clarify the relationship between phenotype–genotype changes and drug resistances.

In this study, we performed laboratory evolution of *Escherichia coli* under various drug treatment conditions to obtain resistant strains. For each obtained drug-resistant strain, transcriptome and genome re-sequencing analyses were performed to identify fixed mutations and gene expression changes. Furthermore, we analysed how the acquisition of resistance to one drug changes the resistance and susceptibility to other drugs. By integrating these data and using a simple mathematical model, we succeed to quantitatively predict resistances to various drugs based on the gene expression levels of a small number of genes. The phenotype–genotype relationship in resistant strains is analysed to elucidate the contribution of the fixed mutations to the drug resistance.

## Results

### Laboratory evolution of antibiotic-resistant *E. coli* cells

**Figure 1:**
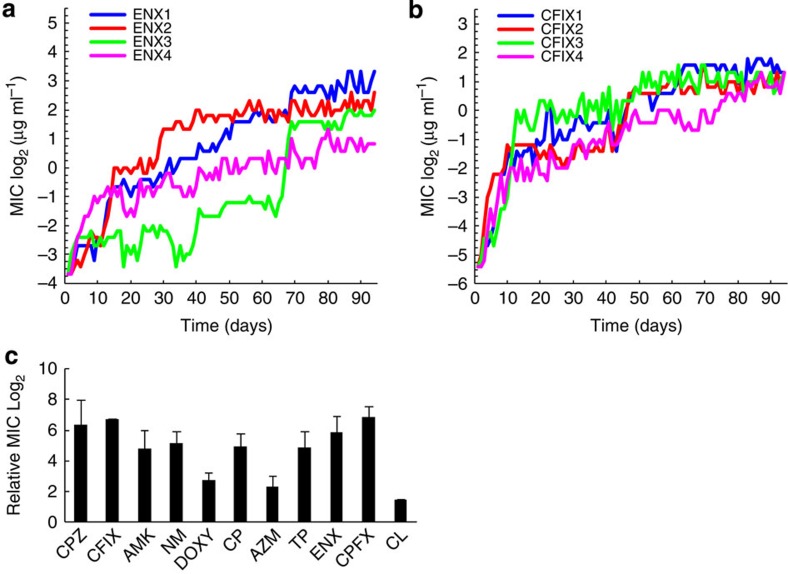
Laboratory evolution of antibiotic resistance. (**a**,**b**) The time courses of the increase in MIC for ENX and CFIX in 90 days experimental evolution, respectively. Day 0 corresponds to the parent strain before evolution. Four parallel series of experiments were performed. (**c**) The increase in MICs for 11 antibiotics used for experimental evolution. The log_2_-transformed averages of relative MICs of resistant strains to the parent strain (day 0) for each antibiotic are presented. The error bars represent the standard deviation from four parallel-evolved resistant strains.

We selected 11 antibiotics that cover a wide range of action mechanisms, including drugs that disrupt cell wall synthesis, protein synthesis, folic acid biosynthesis and DNA replication (Table 1). *E. coli* MDS42 cells were cultured in M9 synthetic medium with eight different concentrations of drugs and were propagated daily from a well containing the highest drug concentration possible, in which cells were able to sustain growth (see Methods for details). To evaluate the reproducibility of the evolutionary pathways, for each antibiotic, four independent culture lines were propagated in parallel. After 90 days propagation, significant increases in minimum inhibitory concentrations (MICs) were observed in the culture series of all 11 antibiotics except colistin (Fig. 1; all time courses are presented in Supplementary Fig. 1). In addition to these cultures, we observed 90 days propagation of two independent culture lines under the antibiotic-free condition, where all other conditions were identical to the other culture lines, as control. For all resistant strains, we confirmed drug resistances after cultivation for at least 30 generations in the absence of the drug, indicating that the phenotypes of drug resistance were stably memorized.

### Quantification of cross-resistance and hyper-susceptibility

**Figure 2:**
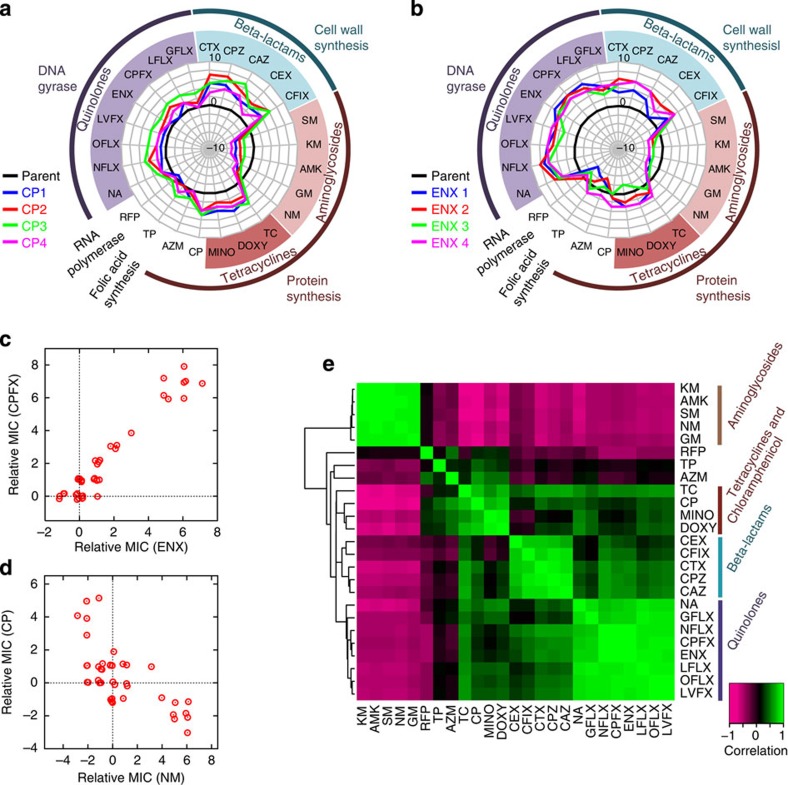
Cross-resistance and hyper-susceptibility between antibiotics. (**a**,**b**) Changes in MICs for other antibiotics in CP and ENX resistant strains, respectively. The radial axis depicts the log_2_-transformed relative MIC to the parent strain. The black thick line indicates MICs of the parent strain, and the coloured thick lines indicate relative MICs of four parallel-evolved resistant strains. (**c**,**d**) The relationships between the MICs of resistant strains for (**c**) ENX and CPFX and (**d**) NM and CP. Red circles represent the relative MICs of all but colistin-resistant strains to the parent strain for the antibiotics depicted in the axes. The data points have been slightly randomized to avoid overlapping of points. (**e**) Pearson’s correlation coefficients for all pairwise antibiotic combinations. The order of antibiotics was determined by hierarchical clustering. Bottom left shows the colour panel indicator of the correlation coefficient.

To explore how the resistance acquisition to one drug changes the resistance and susceptibility to other drugs, for each obtained resistant strain, we measured the MICs of the 25 antibiotics shown in Supplementary Table 1. Figure 2a,b show changes in the MICs of various drugs for chloramphenicol (CP) and enoxacin (ENX)-resistant strains, respectively. The results demonstrated that these antibiotic-resistant strains generally exhibited significant changes in the MICs of multiple drugs (all MIC data are presented in Supplementary Fig. 2). The spectra of MIC values were generally similar among strains resistant to a given drug, suggesting that independently evolved resistant strains reached a similar phenotype. Supplementary Figure 2k shows the MICs of the control strains obtained by serial propagations under the antibiotic-free condition and demonstrates that MICs were unchanged when the cells were cultured without antibiotics.

Cross-resistance is a phenomenon where the acquisition of resistance to a specific drug causes resistance to another drug simultaneously. As expected, cross-resistance to drugs with the same action mechanisms was widely observed. For example, strains resistant to ENX also showed resistance to other quinolone antibiotics (Fig. 2b). Cross-resistance to drugs with other action mechanisms was also observed, as in the case of the beta-lactam resistance exhibited by the CP- and ENX-resistant strains. One possible cause of the observed cross-resistance is that the resistance acquisitions to these drugs shared the same mechanisms. Interestingly, in some cases, resistant strains to a drug became more susceptible than the parent strain, a phenomenon called hyper-susceptibility. For example, CP-resistant strains showed cross-susceptibility to aminoglycosides such as neomycin (NM) and amikacin (AMK).

To further study the interplay among the mechanisms for drug resistances and susceptibility, the relationship between the MICs of two drugs was analysed. Figure 2c shows the relationship between the MICs of ENX and ciprofloxacin (CPFX). The clear positive correlation suggests that these drugs share the same mechanism of resistance. In contrast, the MICs of CP and NM in Fig. 2d showed a significant negative correlation (Pearson’s correlation coefficient *R*=−0.71; *P*<10^−6^), indicating that there is a trade-off between resistances to these drugs.

To represent the overall relationship among drug resistances, we plotted Pearson’s correlation coefficients for all pairwise drug combinations, in which the order of the drugs was determined by hierarchical clustering using the similarity of correlation coefficients as the distance measure (Fig. 2e). The green clusters close to the diagonal line represent groups of drugs showing cross-resistance, and the analysis indicated that drugs with same action mechanisms generally exhibited cross-resistance. Interestingly, aminoglycoside drugs showed a negative correlation to other drug classes, meaning that when *E. coli* cells acquired resistance to aminoglycoside drugs, these resistant strains generally became more susceptible to other drugs than the parent strain, and vice versa. These results are consistent with several previous works that analysed antibiotic resistance by *E. coli* experimental evolution^7^,^21^,^22^. For example, the study by Lázár *et al.*^21^ analysed the cross-resistance network, in which not only the cross-resistances between drugs belonging to the same groups, but also those between drugs in different functional groups, such as quinolones and beta-lactams, were also demonstrated. In addition, Imamovic and Sommer^22^ investigated collateral sensitivity network using *E. coli* to develop drug cycling protocols, in which the trade-off of resistances between aminoglycosides and drugs in other functional groups were demonstrated.

### Prediction of antibiotic resistance from transcriptome data

**Figure 3:**
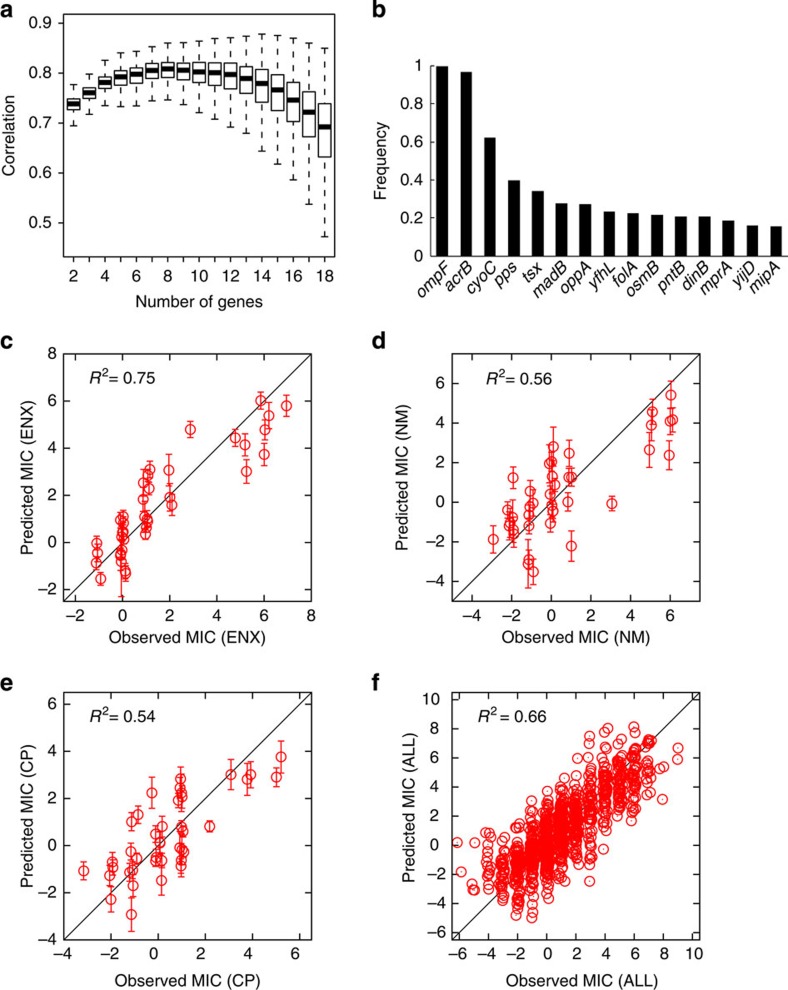
Prediction of antibiotic resistance using transcriptome data. (**a**) Box plot of prediction accuracy as a function of the number of genes, *N*, used for the fitting. The prediction accuracy was quantified by the correlation coefficients between predicted MICs and observed MICs of the test data. (**b**) Frequency of genes selected in GA trials in cases with *N*=8. Randomly generated 10,000 sets of test data and training data were used to calculate the frequency. Comparisons between observed and predicted MICs for (**c**) ENX, (**d**) NM, (**e**) CP and (**f**) all data were calculated by fitting using the following eight genes: *acrB, ompF, cyoC, pps, tsx, oppA, folA* and *pntB*. Only test data not used for the fitting are plotted. The error bars in the *y* axis represent the standard deviation of predicted MICs calculated from 10,000 different sets of test data and training data. The data points have been slightly randomized to avoid the overlapping of points.

The results shown in Fig. 2 demonstrated that an acquisition of resistance to one drug drastically changed resistance and susceptibility to other drugs, which suggests that the phenotypic changes that occurred in the resistant strains were not always restricted to specific factors, such as modification of the drug target protein structure, but instead caused changes in several intra-cellular properties. In the present study, we hypothesized that such phenotypic changes are represented by changes in the gene expression profile, which we tried to extract from transcriptome data obtained by microarray analysis. For the transcriptome data, all resistant strains were cultured in synthetic medium without drug addition to standardize the culture condition among the strains (all transcriptome data are presented in Supplementary Data 1). To examine the contribution of the gene expression changes to the antibiotic resistances, we constructed a simple mathematical model to predict the resistances using the obtained gene expression profiles. Here, we assumed that the drug resistances quantified by the MICs are determined as a function of gene expression levels and neglected any direct effect of the mutations on the drug resistance. Furthermore, for simplification, we neglected non-linear effects and cross terms of the gene expression changes. Thus, we assumed the following simple linear model to predict the MICs by the expression levels of *N* genes:


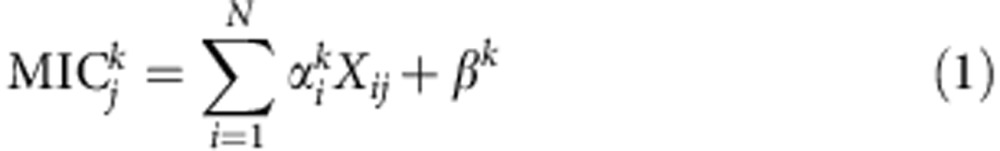



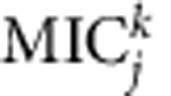
 is the log_2_-transformed relative MIC of the *j*th strain for the *k*th antibiotic, *X*_*ij*_ is the log_10_-transformed expression level of the *i*th gene in the *j*th strain after standardization to zero mean and unit variance, and 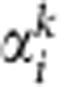 and *β*^*k*^ are fitting parameters. The number of genes in *E. coli* is around 4,000, which is much larger than the number of MIC data. Thus, when we use all genes for the fitting, overfitting is inevitable and leads to a meaningless prediction of the MICs. To avoid overfitting and to seek the number of genes with the highest predictive accuracy, we used the cross-validation method, in which the MIC data were separated into training data used for the parameter fitting and test data used to verify the prediction accuracy. When *N* was large, the prediction accuracy for the test data became small due to overfitting; when *N* was small, the linear combination of genes was insufficient to represent changes of the MICs and the prediction accuracy became small. In this analysis, the appropriate gene sets were searched using a genetic algorithm (GA) with a fitness function based on the prediction accuracy of the test data (see Methods for details). We found that *N*=8 offered the highest prediction accuracy on average (Fig. 3a). This conclusion was based on a random selection of training and test data sets. We iterated the GA fitting using thousands of sets of training and test data, and the frequency of genes selected by these GA trials is shown (Fig. 3b). The genes *acrB* and *ompF* were selected in almost all trials (frequency is close to 1), indicating that their expression changes provided the most relevant information for predicting MIC changes. Figure 3c–f show the prediction accuracy of the linear model using eight genes frequently selected in the GA trials: *acrB, ompF, cyoC, pps, tsx, oppA, folA* and *pntB*. In this analysis, for each drug, the coefficients 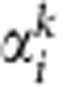 of the eight genes were obtained by fitting the training data, and the plotted data are test data that were not used for the fitting. The estimated MICs agreed well with the observed ones, indicating that the linear model can represent the phenotypic changes that acquire drug resistance using a small number of genes.

To verify whether our linear model simply discriminates the resistant strains from non-resistant strains or it can quantitatively predict the resistances of non-resistant strains also, we evaluated the prediction accuracy after excluding groups of strains evolved to a given class of antibiotics (Supplementary Fig. 3). For example, to evaluate the prediction accuracy for ENX resistance shown in Supplementary Fig. 3a, the data of evolved strains under quinolone antibiotics (ENX, CPFX) were excluded, and the remaining data were randomly separated into training and test data sets. Then, the parameters were fitted using the training data sets, and predicted and observed MICs of test data sets were plotted. Furthermore, by using the fitted parameters, MICs of the excluded strains were also estimated. These results demonstrated that, although the prediction accuracy slightly decreased upon the exclusion of corresponding resistant strains, quantitative predictions were possible by using only cross-resistance and hyper-susceptibility data.

**Figure 4:**
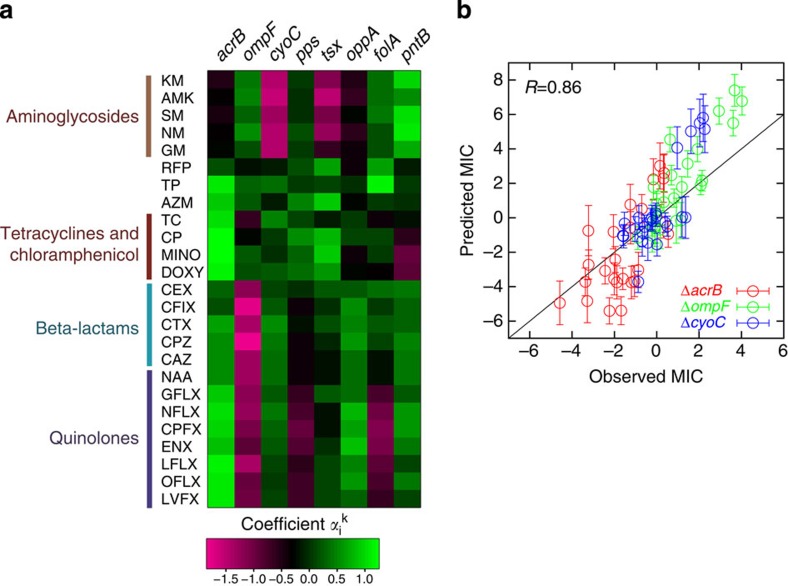
The contribution of the expression levels of eight genes on antibiotic resistance. (**a**) The coefficients 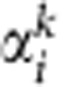 for each antibiotic obtained in the fitting shown in Fig. 3. (**b**) Comparisons between observed and predicted MICs for Δ*acrB*, Δ*ompF* and Δ*cyoC*. For each deletion strain, MICs for the 25 drugs presented in Supplementary Table 1 were quantified. For the prediction, the expression level of the disrupted gene was set to background level. The error bars represent the standard deviation of predicted MICs calculated from 10,000 different sets of test data and training data. The data points have been slightly randomized to avoid the overlapping of points.

The obtained 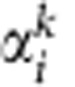 of the above eight genes for each antibiotic are shown (Fig. 4a) and provide information on the cellular processes responsible for the drug resistance acquisition. *acrB* encodes a subunit of a well-characterized multi-drug efflux pump that forms a complex with AcrA and TolC^23^. The increase of *acrB* expression is known to contribute to resistance against various drugs^24^,^25^. The estimated 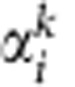 of *acrB* were positive for all investigated drugs except aminoglycosides, which suggests that the upregulation of *acrB* contributed to the resistance. *ompF* encodes an outer membrane porin protein, which allows for the passive diffusion of small molecules^26^. It is known that a decrease of *ompF* expression results in decreasing drug uptake, which leads to drug resistance^26^. The estimated 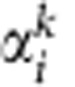 of *ompF* were relatively large negative values for beta-lactam and quinolone drugs, suggesting that the observed resistance in the obtained resistant strains was mainly explained by the *ompF* downregulation. In aminoglycoside-resistant strains, the 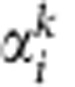 of *cyoC*, which encodes the subunit of cytochrome bo terminal oxidase, were also relatively large negative values, suggesting that the downregulation of this gene or related genes contributed to aminoglycoside resistance. Because the analysis was based on correlations between gene expression changes and resistant acquisition, we could not necessarily conclude a direct causal relationship. To verify the prediction of our model, we constructed deletion strains of the *acrB*, *ompF* and *cyoC* genes, and then quantified the changes of the MICs of multiple drugs. The observed MICs of these deletion strains agreed well with the predicted MICs (Fig. 4b), in which the parameter values were obtained from the fitting of the resistant strains, and the expression levels of the deleted genes were set to background level. This result demonstrated that, at least for these three genes, our model could quantitatively represent the contribution of the gene expression changes to drug resistance.

The ability to predict resistance levels quantitatively does not necessarily mean that our linear model can predict cross-resistance and collateral sensitivity interactions among drugs. When positive or negative correlations between predicted resistances by the model are observed, one possible cause is non-trivial correlations among gene expression levels in the resistant strains, while another possibility is that cross-resistance and collateral sensitivity are embedded in the coefficients 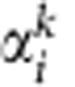 of the linear model. A direct test to discriminate between these two possibilities is to check the correlations among predicted antibiotic resistances by using randomly generated expression levels without any correlations instead of experimental data. Thus, we analysed the correlations of antibiotic resistances obtained by the fitted parameters shown in Fig. 4a with randomly determined expression values of the eight genes. To do this, first, we set the maximum and minimum expression levels for each gene from the experimentally obtained expression levels of all resistant strains. Then, we created 100 sets of artificial expression profiles of the eight genes, in which each expression level was randomly determined by a uniform distribution between the maximum and the minimum expression levels. For each of the random expression profiles, we calculated the resistances to 25 antibiotics using the fitted parameters in Fig. 4a, and then the correlation of resistances between all the possible combinations of two antibiotics were obtained using the artificial data. Supplementary Figure 4b shows correlation coefficients for all pairwise drug combinations obtained by the randomly generated expression levels, and Supplementary Fig. 4c represents the relationship between correlation coefficients obtained from the experimental and random expression data. The predicted pairwise correlations by random expression profiles agreed well with the experimentally obtained correlations, which strongly suggested that the cross-resistances and collateral sensitivity interactions are not generated from specific expression patterns in the resistant strains, but rather are embedded in the fitted parameters of the linear model.

### Mechanism of trade-off between antibiotic resistances

In the aminoglycoside-resistant strains, the 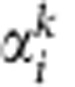 of *cyoC*, whose product is involved in the electron transfer system (ETS)^27^, were relatively large and negative, suggesting that the downregulation of this gene contributed to the aminoglycoside resistance. In fact, gene expression analysis revealed that the *cyoC* gene involved in the ETS was significantly downregulated in NM-resistant strains (Supplementary Fig. 5a). This result suggested a decrease in ETS activity in these strains, which can subsequently result in a reduced proton-motive force (PMF) across the inner membrane. This correlation between the downregulation of ETS-related genes and aminoglycoside resistance is consistent with previous studies demonstrating that the PMF is required for aminoglycoside uptake^28^,^29^. That is, the tolerant strains acquired their resistance by decreasing aminoglycoside uptake, which was caused by a decrease in ETS activity and PMF. This decrease in the expression of ETS-related genes may relate to the hyper-susceptibility of aminoglycoside-resistant strains described above. Also mentioned above was how the activity of the multi-drug efflux pump AcrA/AcrB/TolC can contribute to various drug resistances. Note that this efflux pump is a proton antiporter, and its activity depends on the PMF^30^,^31^. Thus, the reduction in PMF in aminoglycoside-resistant strains can cause the decrease in AcrB activity, leading to hyper-susceptibility for other drugs. Recently, the trade-offs between the resistance against aminoglycoside and other drugs were demonstrated in the laboratory evolution of *E. coli*^11^, and several mutations in genes relating to the respiratory chain and the proton pump were identified in the aminoglycoside -resistant strains. On the basis of these results, a hypothesis was proposed where the hyper-susceptibility of the aminoglycoside-resistant strains is at least partly caused by changes to the PMF. Our results suggest that the change in the PMF leading to the trade-off in resistances can also be caused by the expression changes of ETS-related genes.

### Mutations fixed in resistant strains

**Figure 5:**
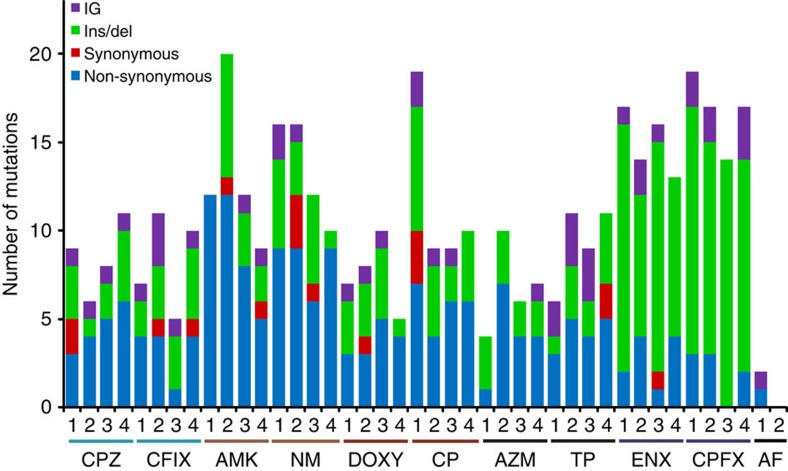
The number of fixed mutations in resistant strains. Mutations were identified using Roche FLX+ and Illumina HiSeq 2000 systems and confirmed by Sanger sequencing (see Methods for details). Blue, red, green and purple bars represent non-synonymous, synonymous, ins/del and intergenic mutations, respectively.

The number of mutations identified in the resistant strains is shown in Fig. 5, and the detailed information is presented in Supplementary Data 2. We analysed the genomic DNA samples using two high-throughput sequencers, Roche FLX+ and Illumina Hiseq. All identified mutations were confirmed by Sanger sequencing (see Methods for details). Less than 20 mutations were fixed in each of the resistant strains. The numbers of fixed mutations were relatively small in the control strains obtained under the antibiotic-free condition, which is consistent with the smaller selection pressure in this condition. We found several genes and gene functions to which mutations were commonly fixed in the resistant strains (Table 2), suggesting contributions by these mutations to the drug resistances.

Mutations in genes relating to the AcrA/AcrB/TolC multi-drug efflux pump were observed in all but the aminoglycoside-resistant strains. Fourteen resistant strains had mutations in *acrR*, a local repressor of *acrAB*^32^. We confirmed that strains with these mutations exhibited significantly higher expression levels of *acrB* (Supplementary Fig. 6), suggesting that these mutations disrupted the repression by AcrR. It is natural to conclude that the mutations in *acrR* were beneficial because of increased expression of *acrB*. In addition, eight non-synonymous mutations were identified in the open reading frame (ORF) of the *acrB* gene, which might suggest that structural changes in AcrB by these mutations can increase the activity of the AcrB efflux pump. To verify the contribution of these mutations on drug resistance, we introduced them into the genome of the parent strain using site-directed mutagenesis^33^. Increases in the MICs of three antibiotics caused by these mutations were relatively small (Supplementary Fig. 7), suggesting that the mutations fixed in the ORF of *acrB* make minor contributions to the drug resistances in comparison with the expression changes of *acrB*. *marR* is another gene known to regulate the expression level of *acrB*, and we found that seven resistant strains had mutations in the ORF or promoter region of *marR*. However, the contribution of the *marR* mutations on *acrB* expression levels was unclear, since five of the seven strains also had mutations in *acrR* or *acrB*.

Mutations in genes related to outer membrane porin protein (Omp) were also observed in various resistant strains. Six mutations were found in *ompR*, which codes a cytoplasmic protein that has been shown to bind upstream of both the *ompF* and *ompC* promoters^34^. Although these mutations might contribute to the expression change of *ompF*, their effects were unclear since the expression levels of *ompF* decreased in most resistant strains regardless of the *ompR* mutations (Supplementary Fig. 8). No further mutations were identified in genes known to regulate *ompF* expression, as according to Regulon DB^35^.

The resistant strains to quinolone DNA gyrase inhibitor (ENX and CPFX) exhibited specific patterns of fixed mutations. In addition to mutations in genes related to the multi-drug efflux pump and porin protein, the quinolone-resistant strains commonly had mutations in genes encoding DNA gyrase (*gyrA*), the target protein of quinolone antibiotics. The contribution of *gyrA* mutations to quinolone resistance has been widely studied^8^. We found that half of the quinolone-resistant strains had mutations in *dinG*, which encodes a putative DNA helicase and is involved in stress-induced DNA-repair, and *mipA*, which encodes a scaffolding protein involved in murein synthesis. The contributions of these mutations to quinolone resistance, however, are unclear. It should be noted that the expression level of *mipA* was significantly downregulated in most quinolone-resistant strains (Supplementary Fig. 9), which might suggest that the disruption of *mipA* function contributed to the quinolone resistance.

Resistant strains to aminoglycosides (AMK and NM) showed a significantly different pattern of mutations from all other resistant strains. In the aminoglycoside-resistant strains, no mutation was found in genes related to *acrB* and *ompF*, for which many mutations were fixed in the other resistant strains. In contrast, aminoglycoside-resistant strains had a variety of mutations in genes related to the respiratory chain and oxidative phosphorylation, including NADH dehydrogenase (*nuo*), cytochrome bo terminal oxidase (*cyo*), and the haem biosynthesis pathway (*hem*). These mutations probably disrupted the activity of the respiratory chain and resulted in a decrease in the PMF. In addition to genes related to respiration, we found that several aminoglycoside-resistant strains had mutations in *cpxA* and in genes related to the sensitive-to-antimicrobial-peptides (Sap) transporter. CpxA is the sensor protein of a two-component signal transduction pathway, which includes CpxR as the cognate response regulator that responds to extracytoplasmic stress^36^. We also found that the expression levels of *cpxA* and *cpxR* were upregulated in the aminoglycoside-resistant strains (Supplementary Fig. 5b). These results are consistent with previous reports that showed mutations in *cpx* genes can constitutively activate the Cpx system, which promotes aminoglycoside resistance by the downregulation of ETS-related genes^37^,^38^,^39^,^40^. The identified mutations and expected expression changes were consistent with previous studies on antibiotic resistance acquisitions. For example, mutations in *acrR* were widely observed in laboratory-evolved and clinically isolated quinolone-resistant strains^21^,^41^,^42^, as observed here. These *acrR* mutations probably contribute to the resistance by upregulating *acrAB* expressions, which is consistent with the prediction of resistances on the basis of the expressions shown in Fig. 3. The mutations in *ompR* were also identified in studies using experimental evolution of *E. coli* under antibiotics^21^,^43^, which can contribute to the resistance through the downregulation of *ompF*, as discussed above. The overlap between mutations identified in our study and previous studies^7^,^11^,^21^,^43^ are presented in Supplementary Data 2.

### Phenotypic convergence and genotypic diversity

Genome re-sequencing analysis demonstrated that mutations in several genes and changes in gene functions were shared among resistant strains. For example, dozens of mutations were found in genes related to the multi-drug efflux pump (*acrAB*), outer membrane porin protein (*ompF*), and respiration chain (*cyo*, *nuo* and *hem* genes), while common changes in the expression of genes related to these functions were suggested to contribute to the resistance according to our simple linear model. For some cases, simple causal relationships between mutations and expression changes can be derived from the observed data. For example, the *acrR* mutations identified in the CP1-, CP2- and CP3-resistant strains caused an upregulation of *acrB* expressions, which gave rise to the observed drug resistances. However, the relationship between fixed mutations and gene expression changes was not always a simple one-to-one correspondence; instead, multiple mutations were suggested to cause similar gene expression changes. For example, the expression of *acrB* was commonly upregulated in resistant strains CP1–4 (Supplementary Fig. 6), whereas mutations in *acrR* were only seen in resistant strains CP1–3. Thus, for the CP4 strain, mutations regulated the *acrB* expression indirectly. One candidate for this regulation was the mutation fixed in *lon*. A previous study showed that some mutations in *lon* stabilize the MarA transcriptional activator, leading to the upregulation of *acrB*^44^. It is, therefore, possible that in the CP4 strain, the *lon* mutation might contribute to the upregulation of *acrB* and CP resistance, unlike in other CP strains where *acrR* mutations were responsible.

A similar diversity of mutations was observed in NM-resistant strains. The expression analysis showed that *cyo* genes encoding cytochrome bo oxidase were commonly downregulated in these strains. In NM1-, NM3-, NM4-resistant strains, mutations were fixed in *cpxA*. As described above, mutations in *cpxA* can activate the CpxA–CpxR signal transduction pathway to downregulate *cyo* genes (Supplementary Fig. 5). However, similar upregulation of *cpxAR* genes and downregulation of *cyo* genes was observed in the NM2 strain even though no mutation was found in *cpxAR* genes. Furthermore, we did not identify any mutations that directly contributed to the observed upregulation of *cpxAR* in NM2, and the mechanism for the upregulation remains unclear, however, we did identify a mutation upstream of the *cyo* operon in NM2, which might contribute to the downregulation of *cyo* genes.

A complex relationship between mutations and gene expression changes was also observed in the downregulation of *ompF* in beta-lactam (CPZ and CFIX)-resistant strains. The expression levels of *ompF* in CPZ-resistant strains were commonly downregulated (Supplementary Fig. 8). In the CPZ1 and CPZ4 strains, mutations were found in the *acrR* and *marR* genes, respectively, both of which were suggested to regulate the expression of *micF*, indicating these mutations contributed to the downregulation of *ompF* and lead to beta-lactam resistance. In contrast, in CPZ2 and CPZ3 strains, no mutation was found to directly regulate the expression of *ompF*. However, we did find in these strains mutations that were fixed in *rfa* genes, which are themselves involved in the lipopolysaccharide biosynthesis pathway^45^. Previous studies have demonstrated that mutations in *rfa* genes can decrease *ompF* and *ompC* expressions, presumably by changing the permeability of the outer membrane^45^,^46^. Thus, these *rfa* mutations might contribute to the CPZ resistance, although the expression level of *ompC* was unchanged in both CPZ2 and CPZ3 strains.

## Discussion

In this study, we performed phenotypic and genotypic analysis of resistant strains obtained by parallel laboratory evolution to various antibiotics. We showed that resistance acquisition to one drug can drastically change the resistance and susceptibility to other drugs. We also demonstrated using a simple linear model that changes in resistance and susceptibility can be predicted by the expression levels of a small number of genes. These same genes could, therefore, be used to describe the phenotypes responsible for the drug resistance and susceptibility. Thus, by significantly reducing the degrees of freedom, we could quantitatively clarify how cells acquire drug resistance and also explain unexpected side effects such as cross-resistance and hyper-susceptibility.

The genes identified frequently selected by the GA trials (Fig. 3b) included several well-known resistance-related genes, such as *acrB* and *ompF*, and also several less-characterized genes. For example, among the eight genes used in the example in Figs 3 and 4, the expression of *oppA* was generally upregulated in quinolones and beta-lactams (Supplementary Fig. 10). *oppA* encodes a peptide binding protein, which is an essential component of the oligopeptide transporter^47^. Some previous studies discussed that the deletion of *oppA* contributes to aminoglycoside resistance^48^. Our discovery may offer some insight on the mechanism responsible. Furthermore, several less-characterized genes, which have never been reported to be related to resistance, such as *yhfL* and *yijD*, were also suggested to relate to the resistance acquisitions, which should be elucidated in future work.

We found many mutations commonly fixed in the resistant strains of a given class of antibiotics, suggesting that these mutations contribute to the resistance acquisition. Such tight interactions between resistance acquisition and mutation fixation might suggest that the resistance, susceptibility and cross-resistance can also be predicted by the genomic sequences, which is consistent with a recent study^21^. Genomic-sequence-based analysis would be complementary to our expression-based approach. Our expression-based analysis also demonstrated that the phenotype–genotype maps were complex and included various mutations that caused similar phenotypic changes. Integrating gene expression and genotype data will provide a better description of the mechanisms responsible for resistance acquisition and unveil the relationship between resistance/susceptibility for several drugs.

Our experimental data indicated that, although different mutations were fixed in resistant strains to the same drug, the expression changes among these strains were similar, suggesting that different mutations can cause a similar antibiotic resistance through common expression changes. We suspect that investigating common resistance acquisition mechanisms, such as HGT, will show similar expression changes for the resistance. Thus, it is importance to compare precise phenotype–genotype comparisons when investigating common resistance acquisition mechanisms, such as HGT.

It should also be noted that the identified mutations can contribute not only to antibiotic resistance directly, but also compensate for any fitness cost associated with the resistance acquisition. In fact, the resistant strains we obtained generally exhibited lower growth rate under the antibiotic-free condition (Supplementary Fig. 11). This result might suggest that mutations that compensate for any observed fitness cost associated with the resistance can also be selected in experimental evolution under antibiotics. In future works, such epistatic interactions among the identified mutations should be investigated to unveil which mutations can help compensate for fitness cost.

The acquisition of drug resistance is a phenomenon that involves changes in various components, including the genome, transcripts and metabolites, meaning a complex interaction network is involved. One possible strategy to understanding such complex dynamics is to analyse large-scale data for each hierarchical layer and then to integrate the separate analyzes to extract the essential components for the drug resistance. Our study is one of the first to show detailed comparisons of drug resistance and susceptibility with the transcriptome and whole-genome sequence, and we succeeded to extract gene expression changes responsible for drug resistance. These results suggest that resistant acquisition to various drugs can be predicted by the expression levels of a small number of genes, which might suggest that there are several dominant ‘pathways’ in expression changes to acquire antibiotic resistance. If so, the prevention of antibiotic-resistant strain may be achievable by inhibiting phenotypic changes, for which complex phenotype–genotype mapping will be necessary.

## Methods

### Bacterial strain and culture conditions

The IS elements-free *Escherichia coli* strain MDS42 (ref. 49) was purchased from Scarab Genomics and used throughout this study. Bacterial cells were cultured in 200 μl modified M9 medium^50^ in 96-well microplates (Corning Inc. 3595) with shaking at 900 strokes per minute on a microplate shaker (TITRAMAX1000, Heidolph Instruments) at 34 °C. All the antibiotics used in this study were purchased from Wako Pure Chemical Industries, Ltd. Antibiotic stock solutions were made by dissolving powder stocks in specified solvents by the manufacturer’s instruction. All antibiotic stocks dissolved in water were 0.2 μm filter-sterilized and stored at −80 °C before use.

### Experimental evolution of antibiotic resistance

Four independent cultures for each antibiotic were propagated in parallel with serial diluted antibiotics that were slightly lower than MICs. The range of antibiotic concentrations used for the evolution experiments were in doubling dilution steps up and down from 1 μg ml^−1^ with three quartile concentrations according to the MICs of each evolving culture line. At a daily transfer, cell growth was monitored by measuring the OD_600 nm_ of each well using the microplate reader 1420 ARVO (PerkinElmer Inc.). We defined a well whose OD_600 nm_ was >0.03 as a well in which cells could grow. Cells calculated to yield an initial OD_600 nm_ of 3 × 10^−5^ were transferred from the well with the highest drug concentration in which cells could grow to new plates with fresh medium and various concentrations of antibiotics. Cells during and after the evolution experiments were stored as glycerol stocks at −80 °C and used for further analysis.

### MIC measurement

Serial dilutions of each antibiotic were made in 96-well microplates using modified M9 medium and stored at −80 °C before use. The range of antibiotic concentrations used for determining MICs were based on doubling dilution steps up and down from 1 μg ml^−1^ as required depending on the antibiotic. We prepared precultures by shaking glycerol-stocked strains in 200 μl of modified M9 medium in 96-well microplates for 23 h at 34 °C with (evolved resistant strains) or without (parent strain) the antibiotics used for the evolution experiments. The precultured cells, calculated to yield an initial OD_600 nm_ of 3 × 10^−5^, were inoculated into each well in freshly thawed MIC plates to a final volume of 200 μl. After 23 h incubation with shaking, the microplates were read at 600 nm using 1420 ARVO (PerkinElmer). The MICs were defined as the lowest concentration of antibiotic that reduced the growth to an OD_600 nm_<0.03.

### Total RNA isolation

We prepared precultures by shaking −80 °C glycerol-stocked parent and evolved resistant strains in 200 μl of modified M9 medium in 96-well microplates for 23 h at 34 °C without antibiotic. The cells precultured were diluted to an OD_600 nm_ of 1 × 10^−4^ into 200 μl of fresh modified M9 medium in 96-well microplates. Then, cultures were grown with shaking at 34 °C to an OD_600 nm_ in the 0.072–0.135 range (equivalent of 10 generations). One hundred and eighty microlitres of exponential cultures were withdrawn rapidly, and cells were killed immediately by the addition of an equal volume of ice-cold ethanol that contained 10% (w/v) phenol. The cells were collected by centrifugation at 20,000 *g* at 4 °C for 5 min, and the pelleted cells were stored at −80 °C before RNA extraction. Total RNA was isolated and purified from cells using an RNeasy micro Kit with on-column DNA digestion (Qiagen) in accordance with the manufacturer’s instructions. The quantity of the purified RNA was determined by the absorbance at 260 nm using NanoDrop ND-2000 (Thermo Fisher Scientific Inc.). The quality of the purified RNA was evaluated using Agilent 2100 Bioanalyzer with an RNA 6000 Nano Kit (Agilent Technologies). Because the quality of the purified RNA was important for accurate estimation of the gene expression level, two to three independent purifications and qualifications of total RNA were performed for each condition. We utilized only purified RNAs that had an RIN (RNA integrity number) of 9.0 or more. The purified RNAs were stored at −80 °C before transcriptome analysis.

### Expression profiling of genes by microarray experiments

Microarray experiments were performed using the custom-designed Agilent 8 × 60 K array for *E. coli* W3110, in which 12 probes were prepared for each gene. One hundred nanograms of purified total RNAs were labelled using the Low Input Quick Amp WT Labeling Kit (Agilent Technologies) with Cyanine3 (Cy3) in accordance with the manufacturer’s instructions. After confirmation of yields (>825 ng) and specific activities (>15 pmol μg^−1^) of the Cy3-labelled cRNAs using NanoDrop ND-2000, labelled cRNAs (600 ng) were fragmented and then hybridized on the microarray for 17 h while rotating at a speed of 10 r.p.m. at 65 °C in an hybridization oven (Agilent Technologies). Washing and scanning of microarrays were performed in accordance with the manufacturer’s instructions. Microarray image analysis was performed using Feature Extraction version 10.7.3.1 (Agilent Technologies). The background corrected intensity values were normalized using the quantile normalization method. The normalized data of microarrays have been deposited in GEO under the accession code GSE59408 and are presented in Supplementary Data 1. In that table, we also present the biological duplicated data of the parent strain, that is, expression data obtained from different cultures of the parent strain. Comparisons of the biological duplication data demonstrated that 99% of the expression ratios were within the range 1/1.35 to 1.35.

### Predicting antibiotic resistance by gene expression levels

Data for the expression levels of genes in the same operon are generally highly correlated, which can disrupt the convergence of the gene selection presented below. Therefore, in each operon, we selected a gene with the highest average expression level among samples to use for the fitting, and discarded the other genes. To use only quantitatively reliable data, genes with low expression levels (<300 a.u. in any strain) were excluded from the following analysis. Furthermore, we excluded data with relatively small expression changes among the resistant and parent strains, since the expression changes of such relatively unchanged genes dominated the experimental errors. These selection criteria left 330 genes for the analysis. The log_10_-transformed expression levels of the screened genes were standardized to zero mean and unit variance. Using the standardized gene expression data and MIC data of the resistant strains, the fitting parameters 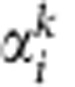 and *β*^*k*^ in equation (1) were obtained. In this analysis, we used a fourfold cross-validation method. That is, the resistant strains were randomly partitioned into four equally sized subgroups; one subgroup was used as the test data set for validation and the remaining three subgroups were used for the fitting. The fitting was performed using the multiple regression implemented in R statistical language.

*N* genes used for the fitting were selected by a genetic algorithm (GA), in which the correlation coefficient between the predicted and observed MICs of the training data sets was used as the fitness function. As an initial population, 1,000 sets of *N* genes were randomly selected, and the fitness of the sets was calculated. Then, gene sets with fitness in the top 5% were selected as the parent sets of the next generation from which mutant sets were generated randomly by replacing a single gene without changing *N*. We iterated 300 cycles of mutant sets and selected gene sets with the highest fitness to obtain sets of *N* genes whose expression levels could represent changes in drug resistances and susceptibility. We repeated the selection of gene sets using 10,000 different training data sets prepared randomly by partitioning the total data set to obtain the frequency of genes selected after the GA, as shown in Fig. 3b. To obtain the results presented in Fig. 3a, we performed GA screenings (300 cycles for each) by changing *N* from 2 to 18.

To evaluate the prediction accuracy in Fig. 3 and Supplementary Fig. 3, we calculated the coefficient of determination, which is defined as


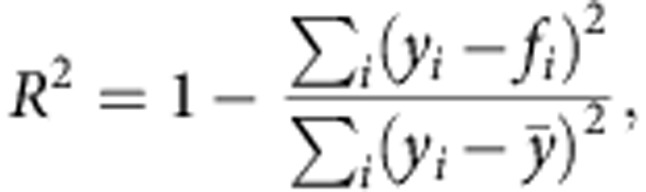


where *y*_*i*_ is *i*th observed data, *f*_*i*_ is *i*th estimated value, and 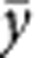 is the average of the observed data. The summation was over all the data obtained by the fitting.

### Genomic DNA preparation

We prepared precultures by shaking stocked strains in 200 μl of modified M9 medium in 96-well microplates for 23 h at 34 °C with (evolved resistant strains) or without (parent strain and two independent cultures under the antibiotic-free condition) the antibiotics used for the evolution experiments. The precultured cells were diluted to an OD_600 nm_ of 3 × 10^−5^ in 10 ml of fresh modified M9 medium in test tubes with or without antibiotic. Cell culture was performed at 34 °C for 23 h with shaking at 150 strokes per minute using water bath shakers (Personal-11, Taitec Co.). After the transfer of 200 μl cultures to microplates, we confirmed that the OD_600 nm_ values of cultures grown in test tubes reached more than 0.2. Rifampicin (final concentration 300 μg ml^−1^) was subsequently added, and the culture was continued for a further 3 h to block the initiation of DNA replication. The cells were collected by centrifugation at 25 °C and 20,000 *g* for 5 min, and the pelleted cells were stored at −80 °C before genomic DNA purification. Genomic DNA was isolated and purified using a Wizard Genomic DNA Purification Kit (Promega) in accordance with the manufacturer’s instructions. To improve the purity of genomic DNA, additional phenol extractions were performed before and after the RNase treatment step. The quantity and purity of the genomic DNA were determined by the absorbance at 260 nm and the ratio of the absorbance at 260 and 280 nm (A_260/280_) using NanoDrop ND-2000, respectively. As a result, we confirmed that A_260/280_ values of all the samples were >1.7. The purified genomic DNAs were stored at −30 °C before use.

### Genome sequence analyses using high-throughput sequencers

Genome sequence analyses were performed with the Roche Genome Sequencer (GS) FLX+ System and Illumina HiSeq System by Takara Bio, Inc. For GS-FLX+ sequencing, a whole-genome shotgun library was prepared according to the manufacturer’s protocol. In this analysis, four DNA samples with different barcodes were applied on a half slide and then sequenced, that is, eight samples were sequenced in a single run, resulting in about 16-fold coverage on average.

An 800 bp paired-end library was automatically generated by the Agilent Bravo Liquid Handling Platform (Agilent technologies) according to the Illumina protocol and sequenced in Illumina HiSeq 2000. In this study, 24 samples with different barcodes were mixed and then sequenced, resulting in about 310-fold coverage on average. The raw sequence data from both Roche FLX+ and Illumina Hiseq systems are available in the DDBJ Sequence Read Archive under accession number PRJDB2980.

In the re-sequencing analysis, we extracted genomic DNA samples from the cell population at the end point of the 90 days experimental evolution without single-colony isolation, since single-colony isolation can fix mutations in a minority of cells and we aimed to identify genotype changes that were fixed in the majority of resistant cells to analyse the phenotype–genotype mapping.

### Alignment of reads and variant call

For the FLX+ data set, we used GS Reference Mapper version 2.6 to align the reads to the reference genome and to generate primary variant calls using default parameters. For nucleotide differences, including point mutations and small ins/del, point mutation and small ins/del calls defined as ‘High-Confidence’ with low total variation percentage (point mutation:<75%, small ins/del:<50%) were removed, as were those defined as ‘All-diffs’ with low coverage (<3) or low total variation percent (<100%). Small ins/dels found in more than four base homopolymer regions were discarded. For structural variations, no candidates were removed at this step.

For the Illumina HiSeq data set, we identified genetic variations according to the computational pipeline developed by the previous study^51^ with slight modification. Briefly, we used paired-end information and mapped HiSeq sequence reads only to best match the MDS42 reference genome sequence using SSAHA2 version 2.5.4 (ref. 52) with default parameters. In this mapping, the quality value of each sequence reads was ignored. For nucleotide differences, including point mutations and small ins/del, point mutation and small ins/del calls with low total variation percentage (point mutation: <75%, small ins/del: <70%) were removed. For potential small ins/del, sequence reads that covered the site were extracted and verified by manually inspecting multiple alignments generated by T-Coffee version 9.03 (ref. 53). The potential nucleotide differences were also validated with *breseq* version 0.24 (ref. 54). To identify large ins/del, previously reported method^51^ was used. Briefly, reads were processed by Quake^55^, and *de novo* assembly was performed by using Soap*denovo*^56^. Then, assembled contigs were mapped on the genome using BLAST^57^, by which candidates for ins/dels were screened. The *de novo* assembly neighbouring candidate sites were aligned to the genome by T-Coffee^53^. The presence of an ins/del was confirmed visually.

Finally, we integrated potential mutations obtained from both platforms and confirmed them using Sanger sequencing of the PCR products. PCR products were checked by agarose gel electrophoresis and purified using the QIAquick PCR Purification Kit (Qiagen). Sanger sequence analyses were performed by Greiner Japan.

In the re-sequencing analysis of the 40 resistant strains and the two culture lines under the antibiotic-free condition mentioned above, we identified 441 and 425 SNP/INDELs in total by Illumina Hiseq and Roche FLX+ analysis, respectively. Among these mutations, 421 were identified by both systems. The 441 and 425 mutations were verified by Sanger sequencing (Supplementary Fig. 12). The results of Sanger sequencing demonstrated that all identified mutations by Illumina Hiseq or Roche FLX+ were true positive, except for one candidate identified by Roche FLX+ only.

Since we sequenced the entire cell population at the end point without single-colony isolation and sequence reads calling minor genomic alterations were ignored, we cannot exclude the possibility that the sample of resistant strains contained minor populations with different mutations that exhibit similar resistances to the major population. However, the existence of such minor populations does not change the results and interpretations of our study, since the present study focused on phenotype–genotype relationships in the major population of resistant strains.

### Markerless allele replacements and MIC measurements

To construct deletion strains of the *acrB* gene, *ompF* gene and *cyoC* operon, and single-nucleotide substitution strains of the *acrB* gene, we introduced mutations into the parent strain by the markerless gene replacement method^33^. Briefly, to construct DNA fragments that had deleted coding regions, upper flanking regions of the start codon were amplified by PCR using genomic DNA of the parent strain as templates with forward primers containing the *Eco*RI site and reverse primers containing overlaps with lower flanking regions of the stop codon. The lower flanking regions were amplified by PCR with forward primers containing overlaps with the upper flanking regions and reverse primers containing the *Kpn*I site. After purification by the MinElute PCR Purification Kit (Qiagen), the PCR products were combined by overlap extension PCR. To construct DNA fragments that introduce single-nucleotide substitutions of the *acrB* gene, DNA fragments were amplified by PCR using genomic DNA of each resistant strain, in which a mutation in the *acrB* gene was identified, with primers containing appropriate restriction sites. Each DNA fragment was purified by the MinElute PCR Purification Kit and then cloned into the suicide plasmid pST76-K^33^ (which was a kind gift from Dr Gyorgy Pósfai, Biological Research Centre of the Hungarian Academy of Sciences, Hungary). After confirmation of DNA fragment sequences by Sanger method, transformation, integration into the parent strain genome, replacement stimulated by double-strand break, and plasmid curing were performed in accordance with a previous method^33^. After construction of mutant strains, corresponding genomic regions were amplified by PCR and then confirmed by Sanger sequencing of the PCR products directly. To evaluate the effects of the mutations on antibiotic resistance, the MICs of these mutants were measured. Briefly, glycerol stocks of the mutants were precultured by shaking in 200 μl of modified M9 medium in 96-well microplates for 23 h at 34 °C without antibiotic. For the deletion strains, the MICs for 25 antibiotics were measured using the same method as mentioned above (see MIC measurement in this section). For the single-nucleotide substitution strains, MICs for cefoperazon, doxycycline, chloramphenicol and trimethoprim were measured three times independently. The step size of the antibiotic concentrations was 0.1 log_2_-transformed on the basis of 1 μg ml^−1^ as required depending on each antibiotic.

## Author contributions

S.S. and T.H. performed the experimental evolution. S.S. performed MIC measurement, microarray experiment, genomic DNA preparation for whole-genome sequencing and construction of mutants. C.F. constructed the linear model and analysed the microarray data and whole-genome sequencing data. S.S. and C.F. designed the experiments and drafted the manuscript. C.F. managed the overall project.

## Additional information

**How to cite this article**: Suzuki, S. *et al.* Prediction of antibiotic resistance by gene expression profiles. *Nat. Commun.* 5:5792 doi: 10.1038/ncomms6792 (2014).

**Accession codes:** The normalized microarray data have been deposited in the GEO database under accession code GSE59408. The raw genomic sequence data have been deposited in the DDBJ Sequence Read Archive under accession code PRJDB2980.

## Supplementary Material

Supplementary Figures and Table Supplementary Figures 1-12 and Supplementary Table 1

Supplementary data 1 Transcriptome data of resistant strains. The log10-transformed expression levels

Supplementary data 2 All identified mutations in the resistant strains.

## Figures and Tables

**Table 1:** List of antibiotics used for experimental evolution.

**Antibiotics name**	**Abbreviation**	**Class**	**Cellular target**
Cefoperazone	CPZ	Cephalosporin, β-lactam	Cell wall
Cefixime	CFIX	Cephalosporin, β-lactam	Cell wall
Amikacin	AMK	Aminoglycoside	Protein synthesis, 30S
Neomycin	NM	Aminoglycoside	Protein synthesis, 30S
Doxycycline	DOXY	Tetracycline	Protein synthesis, 30S
Chloramphenicol	CP		Protein synthesis, 50S
Azithromycin	AZM	Azalide, macrolide	Protein synthesis, 50S
Trimethoprim	TP		Folic acid synthesis
Enoxacin	ENX	Quinolone	DNA gyrase
Ciprofloxacin	CPFX	Quinolone	DNA gyrase
Colistin	CL	Peptide	Cell membrane

**Table 2:** Representative genes in which non-synonymous mutations or ins/dels were commonly fixed in the resistant strains.

**Component**	**Gene**	**Strain**
Multi-drug efflux pump	*acrAB*	CPZ134, DOXY4, CP23, TP234, ENX24
	*acrR*	CPZ1, CP123, AZM23, TP234, ENX14,CPFX234
	*marR*	CPZ4, CFIX4, CP1, AZM23, ENX4
Outer membrane porin	*ompF*	CFIX2, DOXY2, CP34, ENX3, CPFX124
	*ompR*	CFIX23, CP12, ENX2, CPFX3
Electron transfer system	*cyoAB*	AMK24, NM134
	*nuoABCEGMN*	CPZ3, AMK1234, NM1, CPFX1
	*hemAGH*	NM234
Two-component regulatory system	*cpxA*	AMK13, NM134
	*phoQ*	AMK1, TP124
Sap transpoter	*sapACDF*	AMK123, NM13, DOXY24
DNA gyrase	*gyrA*	AZM1, ENX123, CPFX1234
DNA helicase	*dinG*	ENX123, CPFX4
Murein synthesis	*mipA*	ENX3, CPFX124
RNA polymerase sigma unit	*rpoB*	AMK2, NM4, CP34, TP23
	*rpoC*	ENX2, CPFX13
	*rpoD*	CPZ3, TP34
	*rpoN*	CP24
