# Supplementary material for: Prediction of antibiotic resistance by gene expression profiles
Source: Nat Commun. 2014 Dec 17;5:5792. doi: 10.1038/ncomms6792 (PMC4351646; doi:10.1038/ncomms6792)
Supplement: Supplementary Figures and Table — Supplementary Figures 1-12 and Supplementary Table 1 [file ncomms6792-s1.pdf]

Supplementary information

Supplementary Figures

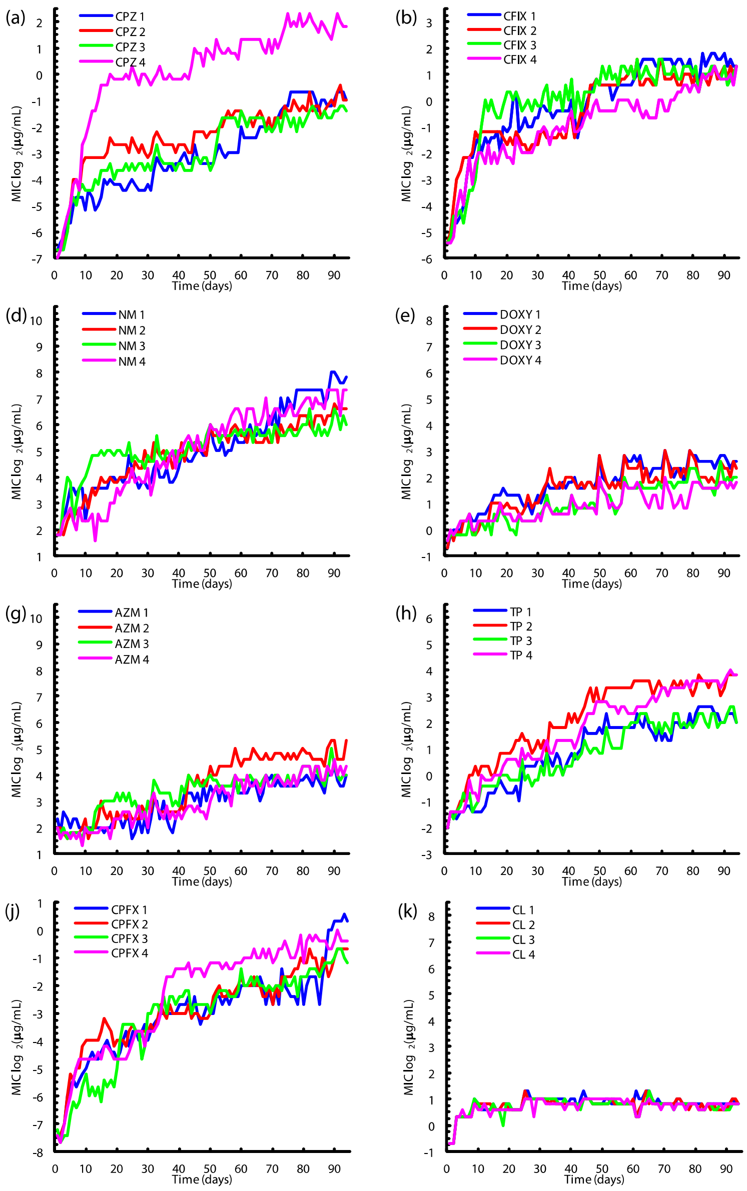

Supplementary Figure 1. The time course of MIC for 11 antibiotics in 90 days of experimental evolution. Day 0 corresponds to the parent strain before evolution. Four parallel series of experiments were performed.

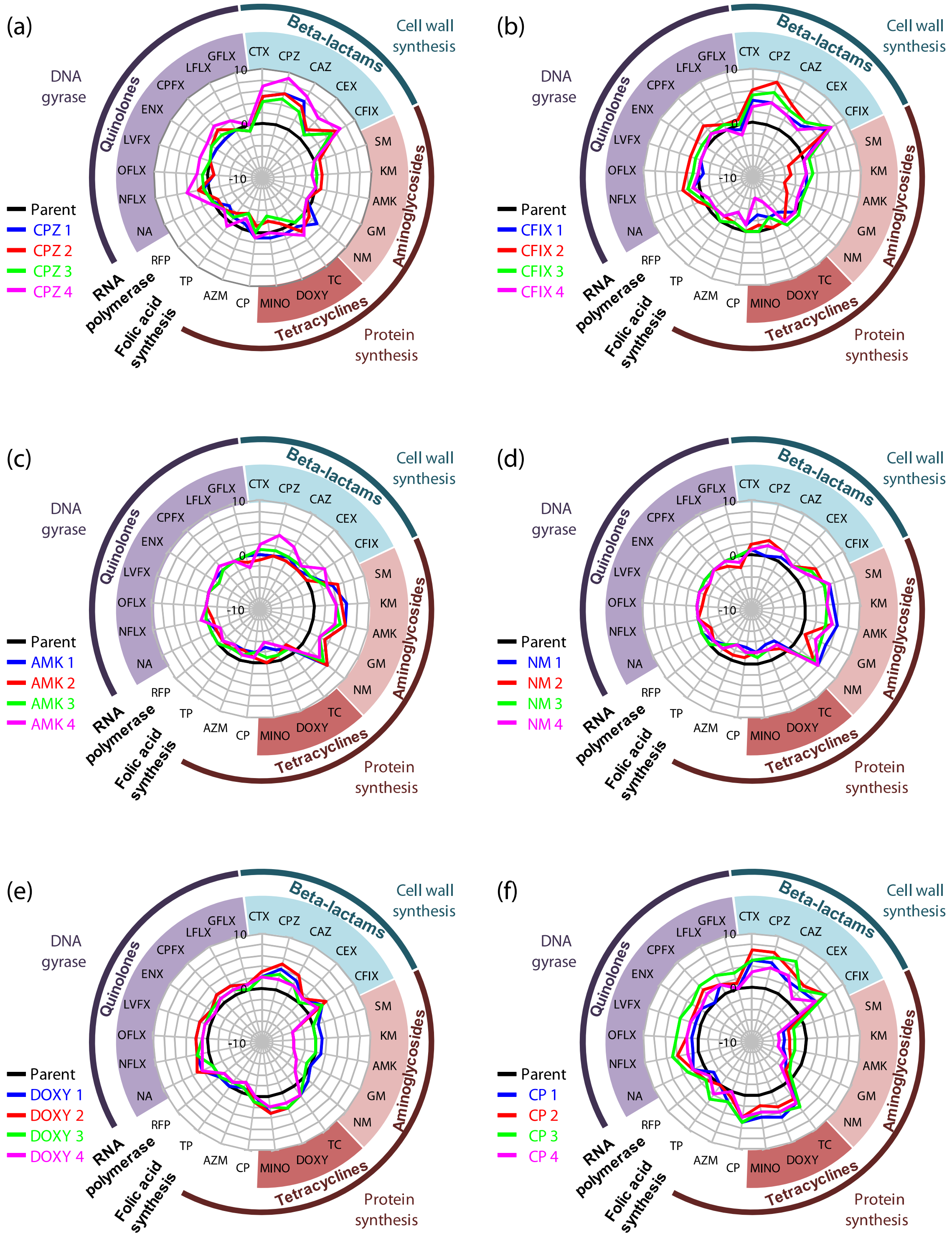
(a)
DNA gyrase
Quinolones
Beta-lactams
Cell wall synthesis
Aminoglycosides
Tetracyclines
Protein synthesis
RNA polymerase
Folic acid synthesis
CTX CPZ CAZ CEX CFIX SM KM AMK GM NM TC DOXY MINO CP AZM TP RFP NA NFLX OFLX LVFX ENX CPFX LFLX GFLX
10
0
-10
Parent
CPZ 1
CPZ 2
CPZ 3
CPZ 4
(b)
Parent
CFIX 1
CFIX 2
CFIX 3
CFIX 4
(c)
Parent
AMK 1
AMK 2
AMK 3
AMK 4
(d)
Parent
NM 1
NM 2
NM 3
NM 4
(e)
Parent
DOXY 1
DOXY 2
DOXY 3
DOXY 4
(f)
Parent
CP 1
CP 2
CP 3
CP 4

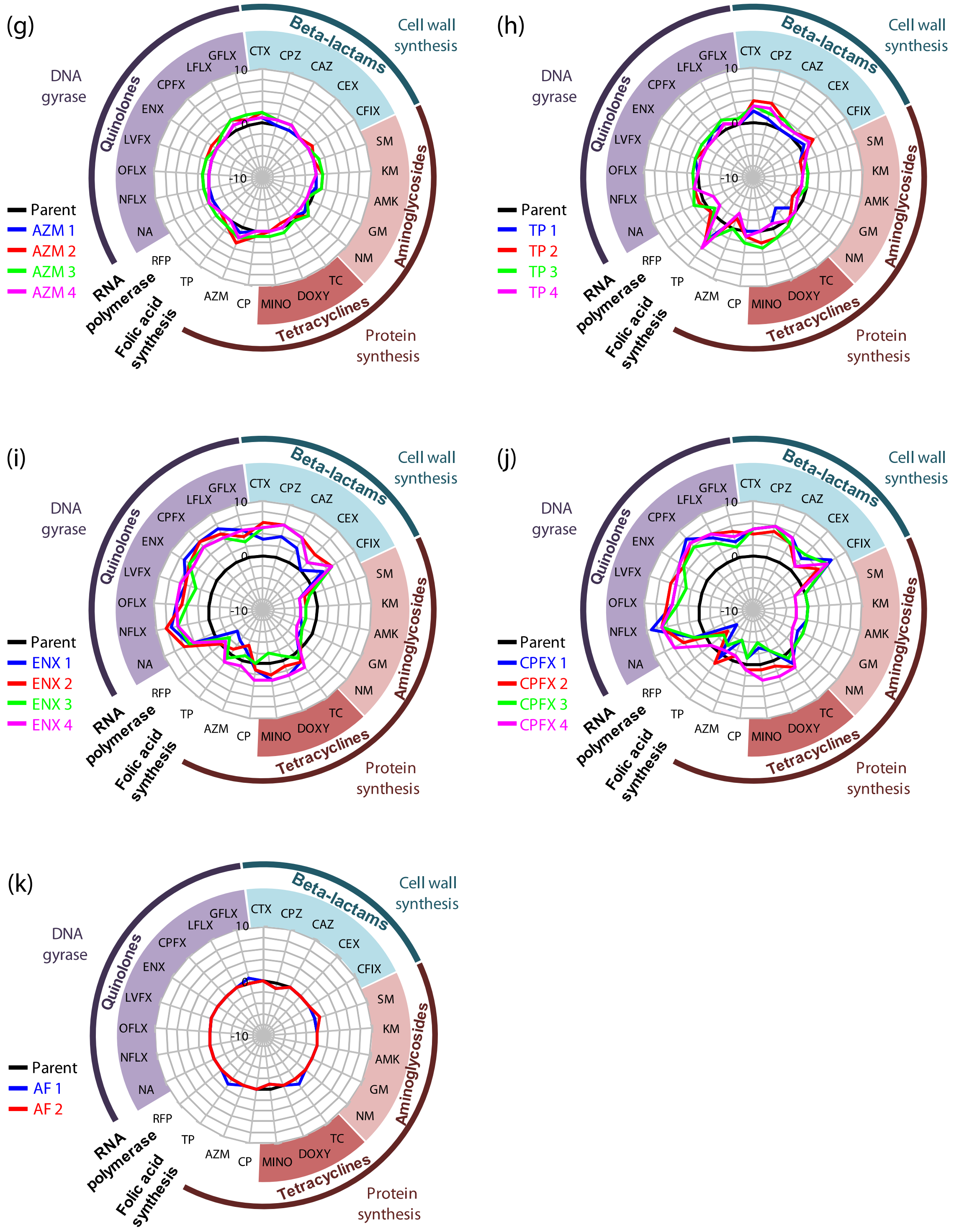

Supplementary Figure 2. Changes in MICs of resistant strains for antibiotics. The radial axis depicts the log_2-transformed relative MIC to the parent strain. The black thick line indicates MICs of the parent strain and the colored thick lines indicate relative MICs of 4 parallel-evolved resistant strains. Graph show the MICs of resistant strains for (a) CPZ, (b) CFIX, (c) AMK, (d) NM, (e) DOXY, (f) CP, (g) AZM, (h) TP, (i) ENX, and (j) CPFX and for control strains obtained under the antibiotic-free condition (k).

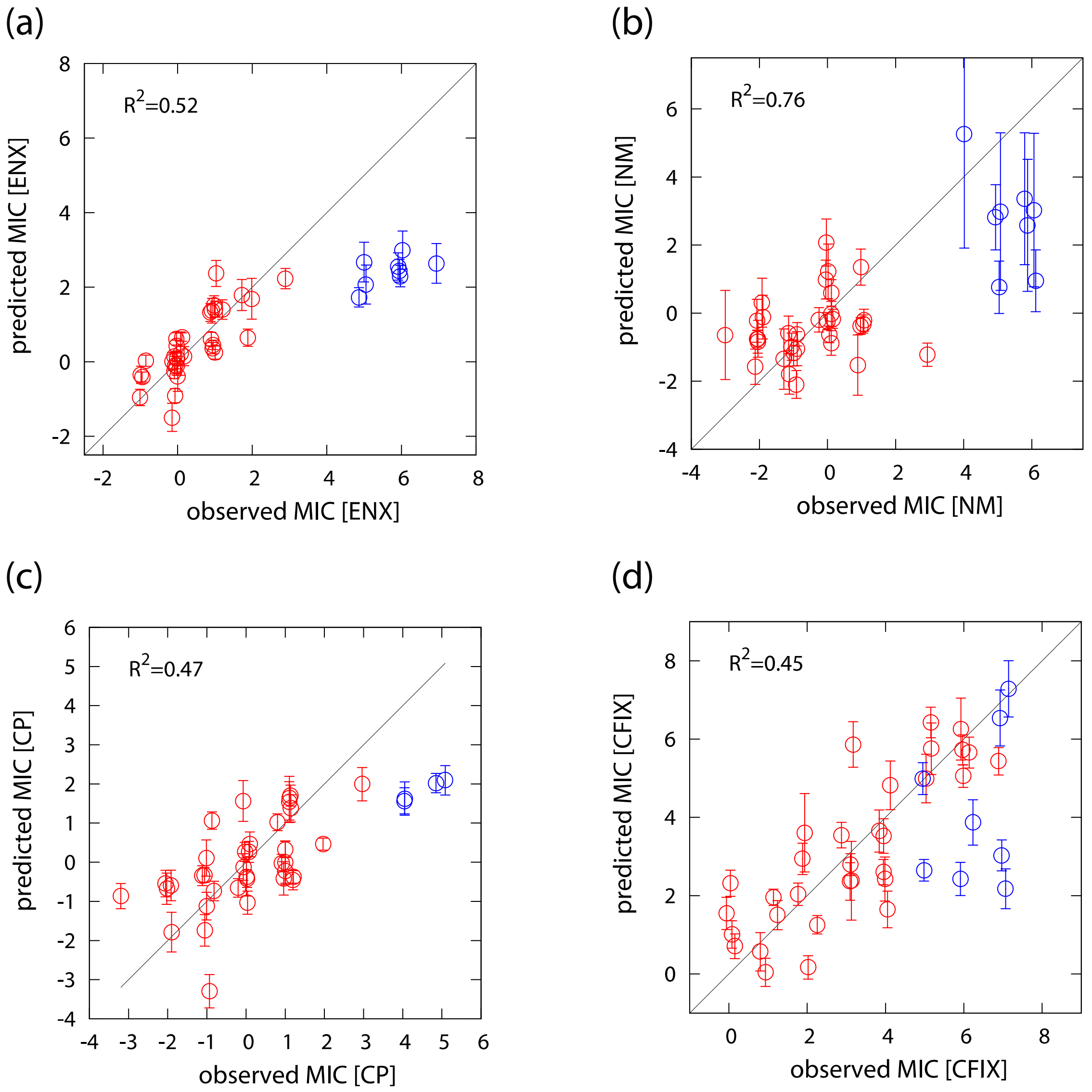

Supplementary Figure 3. Prediction of antibiotic resistance after excluding data of the corresponding resistant strains. The figures represent the prediction accuracies of (a) ENX resistance obtained without using the data of ENX and CPFX resistant strains, (b) NM resistance without NM and AMK resistant strains, (c) CP resistance without CP resistant strains, and (d) CFIX resistance without CFIX and CPZ resistant strains, respectively. The red circles indicate the prediction of test data of non-resistant strains, while the blue circles show the prediction of the resistant strains whose data were excluded from the fitting. The error bars in the y-axis were obtained by predicted MICs calculated from 10,000 different sets of test data and training data. The coefficients of determination (R^2) are also presented.

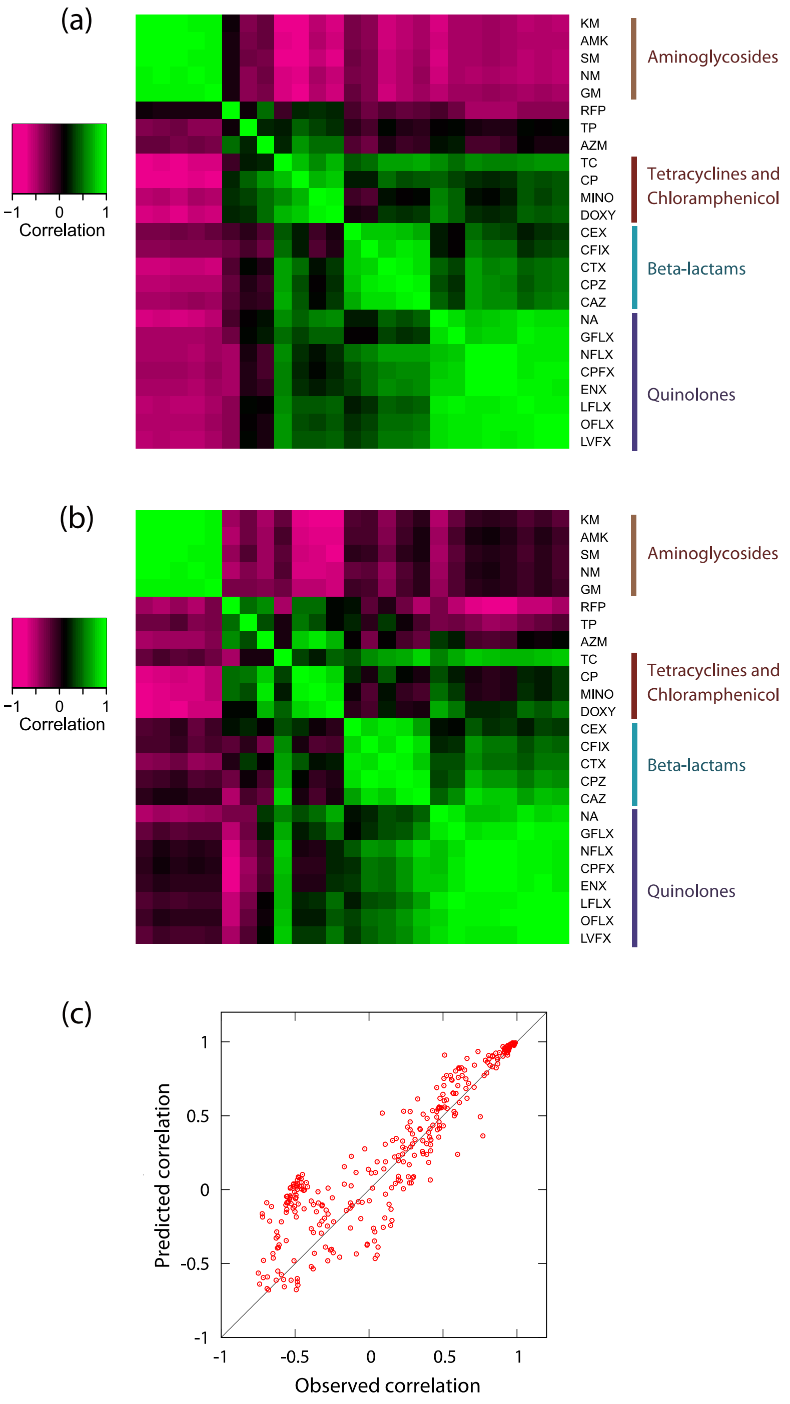

Supplementary Figure 4. Prediction of antibiotic resistance using randomly generated transcriptomic data. (a) Correlation coefficients for all pairwise antibiotic combinations obtained by the experimental data (identical to Fig. 2e; presented for reference). (b) Correlation coefficients calculated using randomly generated expression levels of 8 genes. For the calculation of MICs, the fitting parameters α_i^k and β^k obtained by the fitting of experimental data shown in Fig. 4a were used. The order of antibiotics was identical to that in (a). (c) Comparison between experimentally obtained correlation coefficients and predictions from random expression profiles.

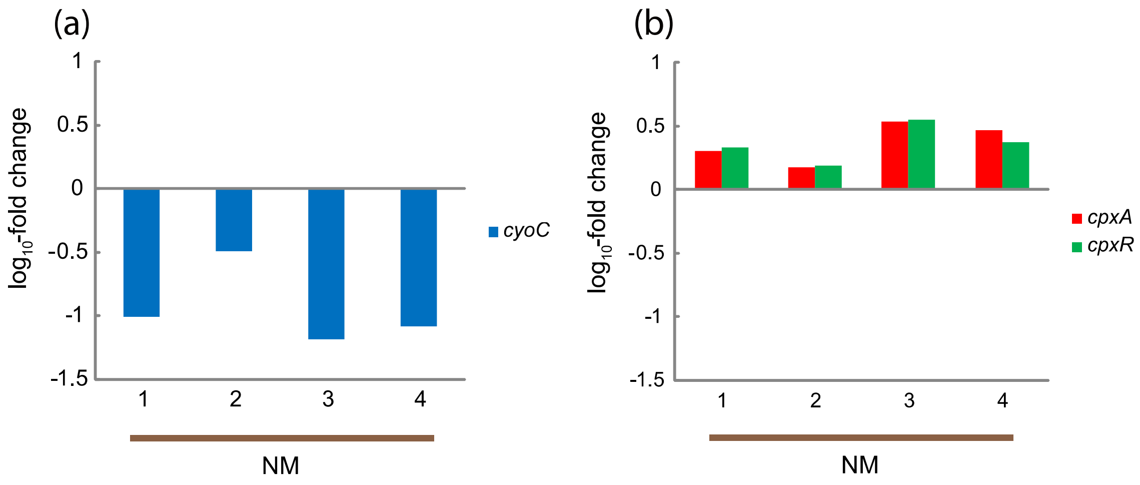

Supplementary Figure 5. Expression changes of *cyoC* and *cpxAR* in NM resistant strains.

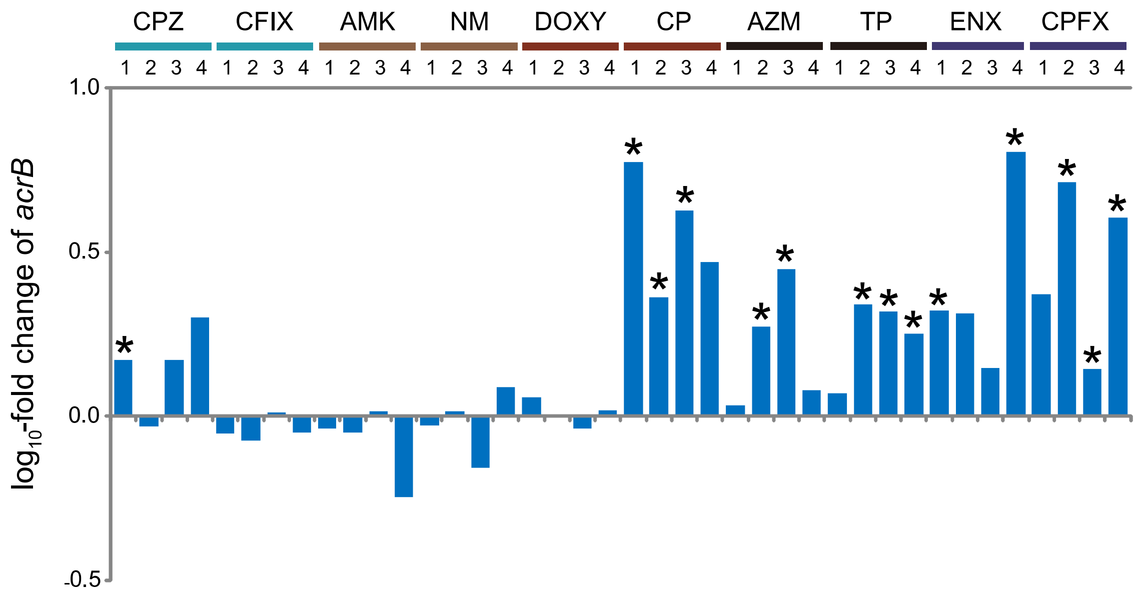

Supplementary Figure 6. Expression changes of *acrB* in resistant strains. The asterisks indicate strains in which an *acrR* mutation was identified.

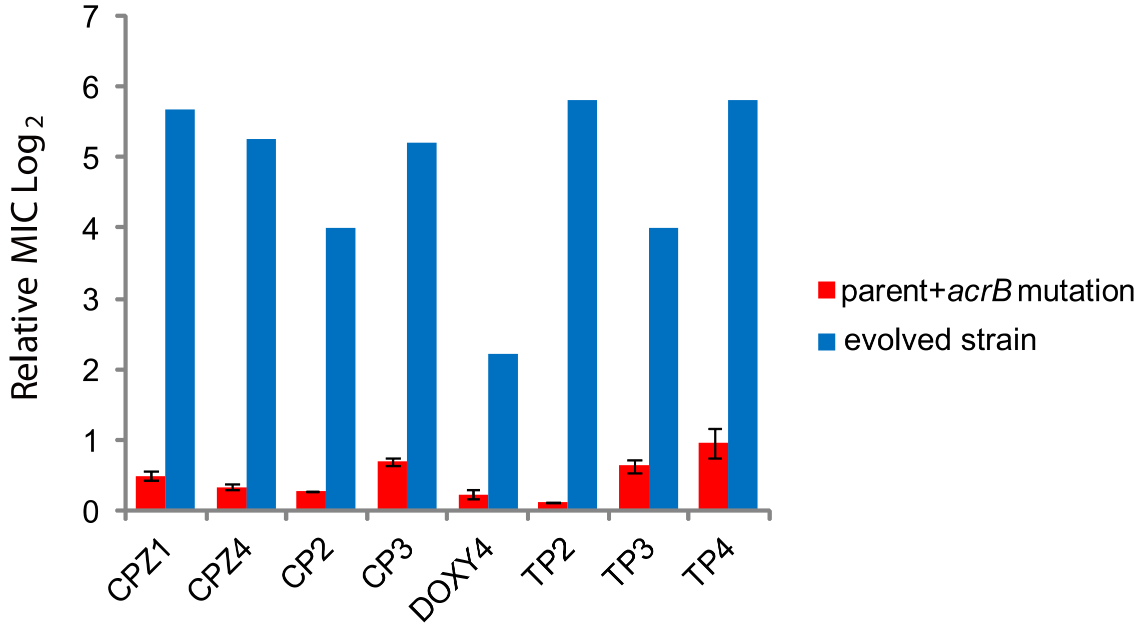

Supplementary Figure 7. Changes in MICs by mutations in the ORF of *acrB*. *acrB* mutations found in 8 resistant strains (CPZ1, CPZ4, CP2, CP3, DOXY4, TP2, TP3, and TP4) were introduced into the genome of the parent strain using site-directed mutagenesis. For each mutated strain, the MIC for the drug used for the corresponding experimental evolution was quantified. The log2-transformed relative MIC of the *acrB* mutant strain to the parent strain is plotted with that of the corresponding resistant strain. Error bars represent the standard deviation of MICs obtained from 3 independent cultures.

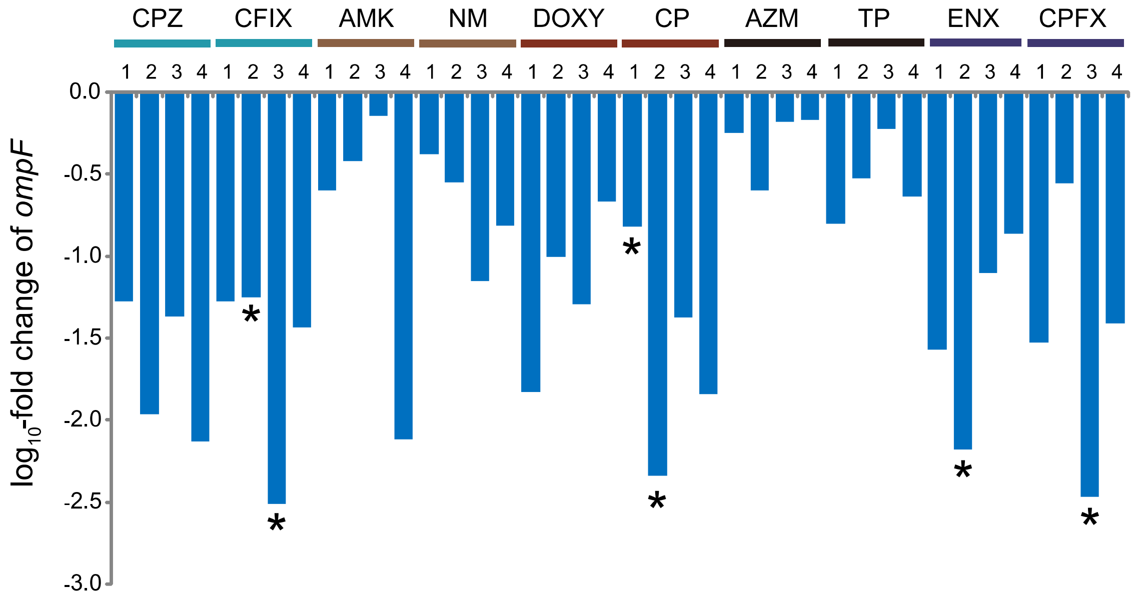

Supplementary Figure 8. Expression changes of *ompF* in resistant strains. The asterisks indicate strains in which an *ompR* mutation was identified.

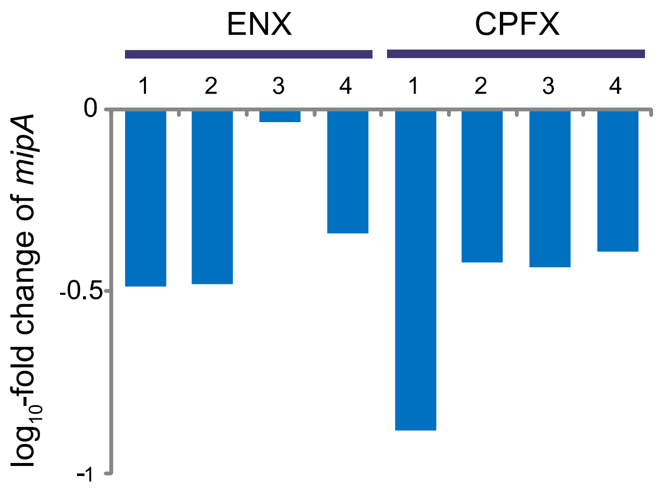

Supplementary Figure 9. Expression changes of *mipA* in ENX and CPFX resistant strains.

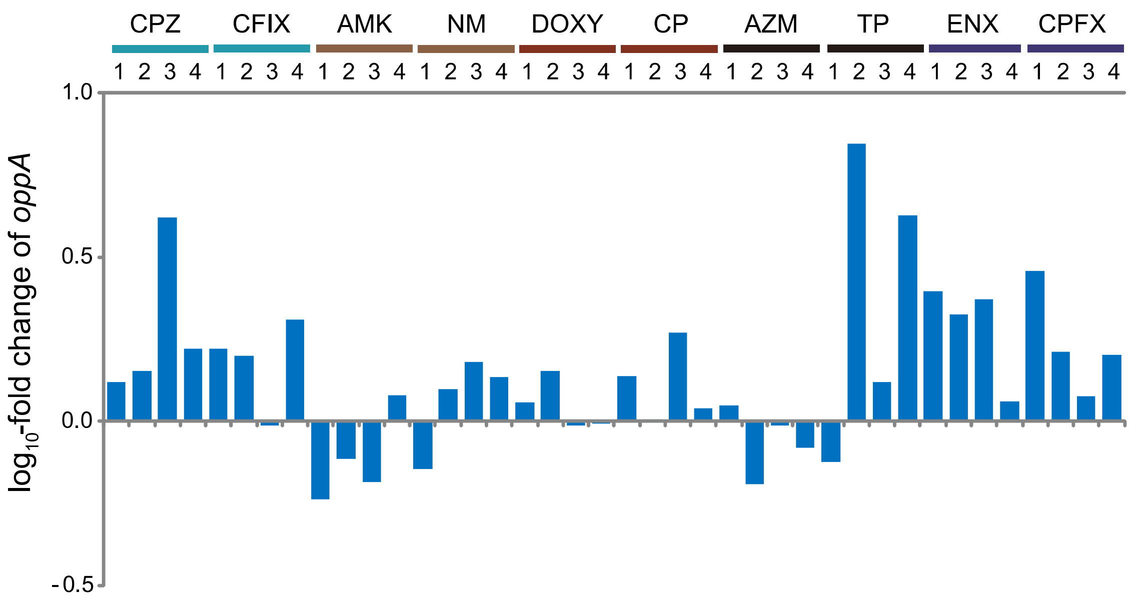

Supplementary Figure 10. Expression changes of *oppA* in resistant strains.

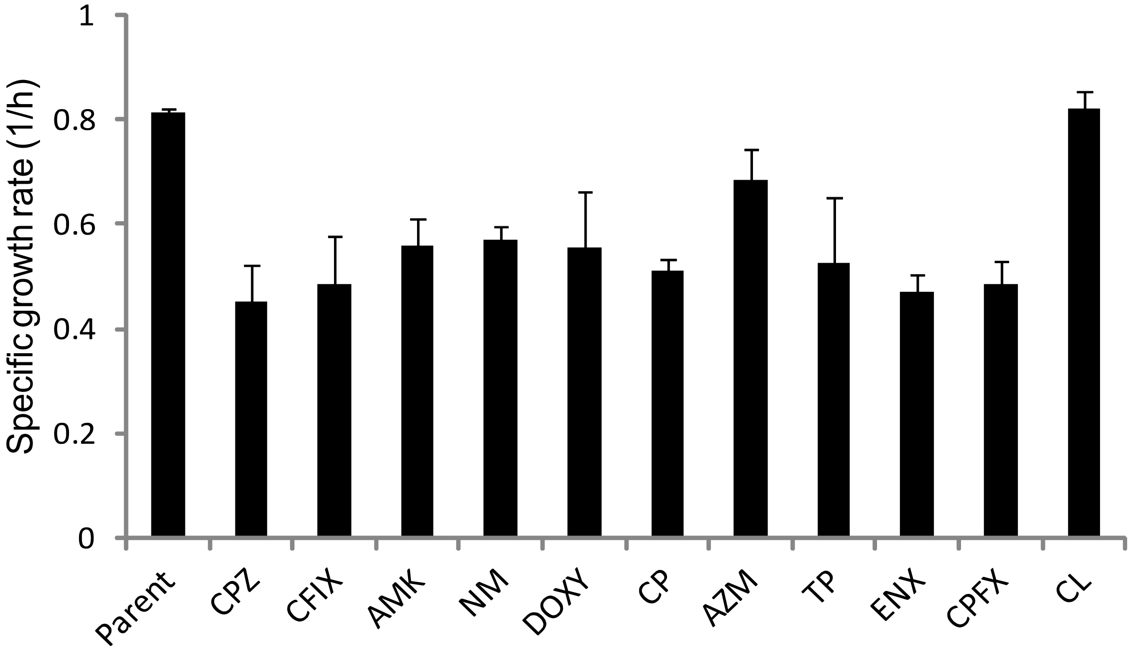

Supplementary Figure 11. Specific growth rate of resistant strains under the antibiotic-free condition. The error bars represent the standard deviation from 4 parallel-evolved resistant strains.

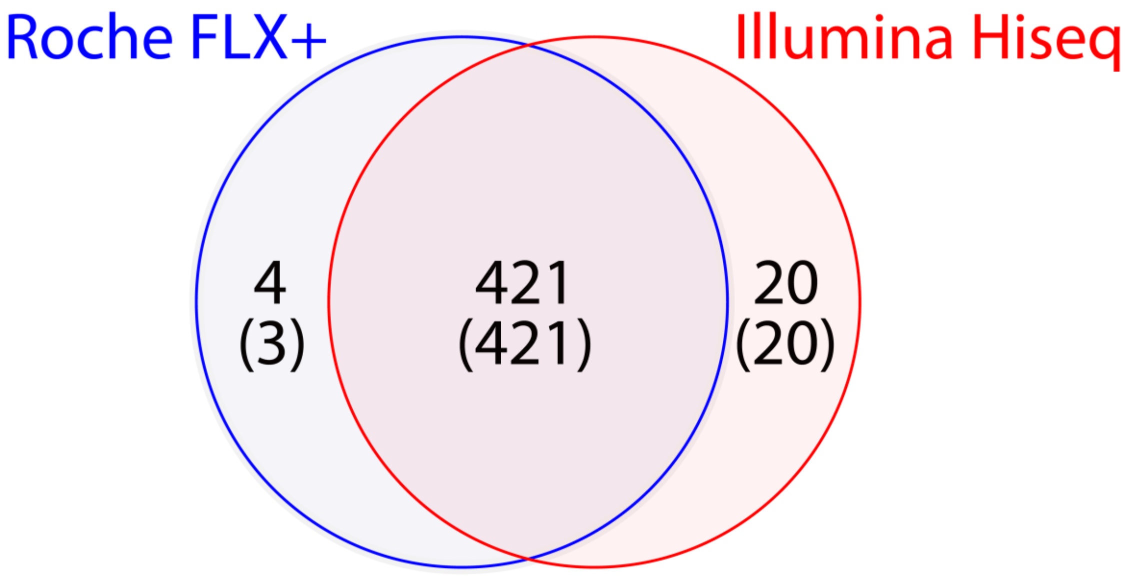

Supplementary Figure 12. Numbers of identified mutations by Illumina Hiseq and Roche FLX+. The numbers in the parenthesis indicate the number of mutations confirmed by Sanger sequencing.

Supplementary Table I: List of antibiotics used in this study

Antibiotics name	Abbreviation	class	Cellular target
Cefotaxime	CTX	Cephalosporin, b-lactam	Cell-wall
Cefoperazone	CPZ	Cephalosporin, b-lactam	Cell-wall
Ceftazidime	CAZ	Cephalosporin, b-lactam	Cell-wall
Cephalexin	CEX	Cephalosporin, b-lactam	Cell-wall
Cefixime	CFIX	Cephalosporin, b-lactam	Cell-wall
Streptomycin	SM	Aminoglycoside	Protein synthesis, 30S
Kanamycin	KM	Aminoglycoside	Protein synthesis, 30S
Amikacin	AMK	Aminoglycoside	Protein synthesis, 30S
Gentamicin	GM	Aminoglycoside	Protein synthesis, 30S
Neomycin	NM	Aminoglycoside	Protein synthesis, 30S
Tetracycline	TC	Tetracycline	Protein synthesis, 30S
Doxycycline	DOXY	Tetracycline	Protein synthesis, 30S
Minocycline	MINO	Tetracycline	Protein synthesis, 30S
Chloramphenicol	CP		Protein synthesis, 50S
Azithromycin	AZM	Azalide, Macrolide	Protein synthesis, 50S
Trimethoprim	TP		Follic acid synthesis
Rifampicin	RFP	Rifamycin	RNA polymerase
Nalidixic acid	NA	Quinolone	DNA gyrase
Norfloxacin	NFLX	Quinolone	DNA gyrase
Ofloxacin	OFLX	Quinolone	DNA gyrase
Levofloxacin	LVFX	Quinolone	DNA gyrase
Enoxacin	ENX	Quinolone	DNA gyrase
Ciprofloxacin	CPFX	Quinolone	DNA gyrase
Lomefloxacin	LFLX	Quinolone	DNA gyrase
Gatifloxacin	GFLX	Quinolone	DNA gyrase
